# OVLI-TA: An Unmanned Aerial System for Measuring Profiles and Turbulence in the Atmospheric Boundary Layer

**DOI:** 10.3390/s19030581

**Published:** 2019-01-30

**Authors:** Sara Alaoui-Sosse, Pierre Durand, Patrice Medina, Philippe Pastor, Marie Lothon, Iuri Cernov

**Affiliations:** 1 Institut Supérieur de l’Aéronautique et de l’Espace (ISAE-SUPAERO), Université de Toulouse, 31400 Toulouse, France; Philippe.PASTOR@isae.fr; 2 Laboratoire d’Aérologie, Université de Toulouse, CNRS, UPS, 31400 Toulouse, France; pierre.durand@aero.obs-mip.fr (P.D.); patrice.medina@aero.obs-mip.fr (P.M.); marie.lothon@aero.obs-mip.fr (M.L.); iuri.cernov@gmail.com (I.C.)

**Keywords:** unmanned aerial vehicle (UAV), turbulence observations, atmospheric boundary layer (ABL), five-hole probe, OVLI-TA

## Abstract

In recent years, we developed a small, unmanned aerial system (UAS) called OVLI-TA (Objet Volant Leger Instrumenté–Turbulence Atmosphérique) dedicated to atmospheric boundary layer research, in Toulouse (France). The device has a wingspan of 2.60 m and weighed 3.5 kg, including payload. It was essentially developed to investigate turbulence in a way complementary to other existing measurement systems, such as instrumented towers/masts. OVLI-TA’s instrumental package includes a 5-hole probe on the nose of the airplane to measure attack and sideslip angles, a Pitot probe to measure static pressure, a fast inertial measurement unit, a GPS receiver, as well as temperature and moisture sensors in specific housings. In addition, the Pixhawk autopilot is used for autonomous flights. OVLI-TA is capable of profiling wind speed, wind direction, temperature, and humidity up to 1 km altitude, in addition to measuring turbulence. After wind tunnel calibrations, flight tests were conducted in March 2016 in Lannemezan (France), where there is a 60-m tower equipped with turbulence sensors. In July 2016, OVLI-TA participated in the international project DACCIWA (Dynamics-Aerosol-Chemistry-Clouds Interactions in West Africa), in Benin. Comparisons of the OVLI-TA observations with both the 60 m tower measurements and the radiosonde profiles showed good agreement for the mean values of wind, temperature, humidity, and turbulence parameters. Moreover, it validated the capacity of the drone to sample wind fluctuations up to a frequency of around 10 Hz, which corresponds to a spatial resolution of the order of 1 m.

## 1. Introduction

Technological advances related to the development of unmanned aircrafts for atmospheric observations make it possible to carry out measurements in areas at very low heights that are difficult to reach by piloted aircrafts, thus covering a wider airspace for collecting meteorological data. The unmanned aerial vehicle (UAV) offers an opportunity to investigate the atmospheric surface layer (ASL) in a way that is complementary to other platforms such as instrumented towers/masts, radiosondes, and piloted airplanes, thereby filling the gap between all available fixed and mobile platforms. 

The ASL is the layer at the bottom of the atmospheric boundary layer (ABL), which directly interacts with the surface of the earth [1]. In general, it is some tens of meters thick [2]. It is characterized by a high variability, both in time (related to the diurnal cycle) and along the vertical; shearing stress is constant in the vertical and the flow is insensitive to the earth’s rotation. Profiles of meteorological parameters, such as temperature, moisture, wind, and turbulence, vary substantially from the surface to the top of the ASL. These follow for example, logarithmic laws or even more complex shapes according to the surface characteristics and turbulence conditions. The two sources of ASL turbulence are friction and buoyancy. The former results from the momentum extracted from the flow, and the latter from the difference in temperature (and, at a lower level, of moisture) between the surface and the air above it. Turbulence is three-dimensional and isotropic, at least for the smaller eddies, which are well described by the “K-41 theory” (Kolmogorov, 1941) on the inertial sub-range and its famous “−5/3 slope” for the spectral energy.

Very high level of ASL knowledge is required for both academic research and practical applications. For example, improving turbulent exchange parameterization in a numerical weather prediction model, or estimating wind turbine wake characteristics require appropriate observations in the ASL. The most important parameters to measure are the temperature, moisture, and wind, at a rate fast enough to capture eddies that significantly contribute to the turbulence energy. Starting from the know-how built up in past decades regarding observations aboard piloted airplanes (see e.g., [3]), and thanks to the miniaturization of sensors and acquisition systems, numerous unmanned aerial systems (UASs) have been developed in recent years all over the world. Similarly to on-board piloted airplanes, many developments were made to characterize the surface overflown, through remote sensing instruments. However, in this paper, we will focus on in situ observations of meteorological parameters and turbulence.

The UAVs were classified according to three main categories, depending on their weight and their payload capacity [4]. The first category regroups all UAVs weighing 10–30 kg, such as for example the Manta, ScanEagle, Aerosonde, and RPMSS (Robotic Plane Meteorological Sounding System); their advantage is their endurance and their highest payload capacity, but they are very expensive and often difficult to operate. The Manta (27.7 kg) and ScanEagle (22 kg), in particular, were used for turbulent flux measurements within terrestrial and marine ABL; wind components and humidity were measured up to 25 Hz and temperature up to 5 Hz [5]. Similarly, ALADINA (Application of Light-weight Aircraft for Detecting IN situ Aerosol) (25 kg) was elaborated for measuring boundary-layer properties, atmospheric particles, and solar radiation, as well as turbulence up to a frequency of ~7 Hz [6].

Secondly, category II includes vehicles that weigh more than 1 kg and less than 10 kg such as the Tempest, the meteorological mini unmanned aerial vehicle (M^2^AV), the NexSTAR, the multipurpose automatic sensor carrier (MASC), and the small multifunction autonomous research and teaching sonde (SMARTSonde). Although these UAVs are smaller, and have less payload capacity and autonomy of flight than the vehicles in category I, they can carry many of the sensors used for wind measurements. In addition to this, their cost is moderate, which makes them more easily deployable. For instance, M^2^AV (6 kg) measures the meteorological wind up to 40 Hz, equivalent to a spatial resolution of 55 cm at an airspeed of 22 m/s, by coupling a GPS and an inertial measurement unit (IMU) with a Kalman filter, and combining data with a five-hole probe for which the calibration had been obtained during a wind tunnel test [7]. M^2^AV and MASC were also used to assess the accuracy and frequency response of a multi-hole probe [8], and finally they can measure fluctuations up to 20 Hz after re-evaluating the pneumatic tubing setup and data acquisition. MASC was also operated for wind energy research [9]. Moreover, the BLUECAT5 (5 kg), developed for turbulence measurements in the ABL, could measure turbulence with a sampling of 60 Hz, by following a profile pattern at loiter radius [10]. However, the circular flight pattern prevents the conversion of time series into spatial scales, except for high rates (small scales) where air sampling can be considered as straight.

Finally category III assembles UAVs that weigh less than 1 kg, for instance the small unmanned meteorological observer (SUMO) and DataHawk, which have limited payload capacity and endurance compared with the two other categories. Their cost and facility of deployment enable small experiments to be carried out from anywhere. SUMO efficiently captures meteorological profiles of wind speed, wind direction, temperature, and humidity [11,12]. Moreover, capabilities and limitations for turbulence observations are described in Reference [13].

Furthermore, different field campaigns have been conducted using UAVs. For instance, SUMO, MASC, and two multicopters participated in the Hailuoto 2017 field campaign in the arctic, in order to increase the understanding of the stable boundary layer by complementing the existing observation systems—like ground-based eddy covariance and automatic weather stations. This large set of data brought insights into the nature of turbulent events that induce rapid warming of layers close to the ice surface [14]. During the CLOUD-MAP campaign, thanks to a great amount of work on sensor integration and calibration/validation, atmospheric sampling of thermodynamic parameters and boundary-layer profiling were done with fixed and rotary wings UASs [15]. Additionally, UAVs are used for specific atmospheric issues as pollution and trace gas monitoring: a six-rotor UAS was used for studying air pollution episode characteristics and influential mechanisms that occurred in Nanjing during the 3–4 December 2017 [16]. On the other hand, three types of UAV including micro aerial vehicles (MAVs), vertical take-off and landing (VTOL), and low-altitude short endurance (LASE) systems, were evaluated to operate three kinds of atmospheric trace gas sensors. The best compromise was given by UAVs, which have wingspans <3 m for payloads <5 kg [17].

The unmanned aerial vehicle OVLI-TA (“Objet Volant Leger Instrumenté–Turbulence Atmosphérique”, that is instrumented light aerial vehicle-atmospheric turbulence) belongs to the second category of UAVs. OVLI-TA was developed at Laboratoire d’Aérologie in Toulouse, France. The purpose was to develop a low cost system able to fly at low altitudes and easy to deploy; that may measure temperature, humidity, wind vector, and observe turbulence within the ABL. One of the unique attributes of the OVLI-TA system with respect to comparable UAVs is that its nose serves as a five-hole probe, which is 3D printed, easy to manufacture, and allows us to install the inertial platform and aerodynamic sensors in the same location. Thus, when solving the 3-component wind equation (see Section 2), even at a rate fast enough to capture turbulent scales, all the terms with angular rotations can be neglected. Whereas, they must be taken into account when using systems based on a multi-hole probe placed at the extremity of a boom. Furthermore, tubing length effects on pressure measurements are eliminated in our system because the transducers can be installed as close as possible to the pressure ports. Regarding UAV performance, our choice was guided by a compromise between a system small enough to be easy to operate, and large enough to allow a payload compatible with a good-quality sensors package and an endurance compatible with atmospheric boundary layer probing. With our system, the autonomy of flight was at least one hour. Moreover, even if OVLI-TA is lighter (3.5 kg) than other category 2 UAVs (e.g., 6 kg for the M^2^AV and 5 to 7.5 kg for the MASC), it permits comparable horizontal resolution. In addition to the hemispherical 5-hole probe situated on the nose of the airplane to measure attack and sideslip angles, there is a Pitot probe, a fast IMU, a GPS receiver, as well as temperature and moisture sensors placed in specific housings. Its cost is affordable, and hence we can risk flying in areas with turbulent conditions. OVLI-TA is mainly a system for profiling the atmospheric boundary layer, it also has the capacity to measure turbulence properly. Several flights were conducted in Lannemezan (France), where there is an equipped 60 m tower that was a reference to our measurements. The drone then participated in the international project DACCIWA (Dynamics-Aerosol-Chemistry-Clouds Interactions in West Africa), in Benin.

This paper gives a technical description of OVLI-TA, and highlights the results of profiling the atmospheric boundary layer obtained during flights in Lannemezan and in DACCIWA. The performance for measuring the mean meteorological parameters was evaluated against the 60 m tower and radiosonde profiles. Furthermore, turbulence observations on the tower and with the UAV were compared through a time–space conversion based on Taylor’s hypothesis applied to the two platforms.

## 2. OVLI-TA Unmanned Aerial Vehicle and Methods

### 2.1. OVLI-TA Airframe Characteristics

**Figure 1:**
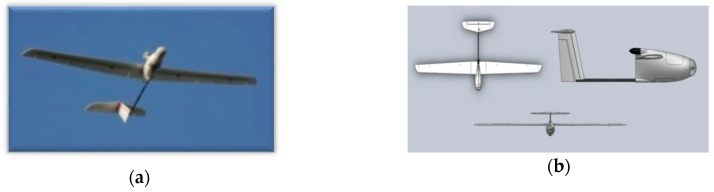
OVLI-TA UAV (**a**,**b**) based on the commercial airborne model Techpod, manufactured by HobbyUAV.

OVLI-TA (Figure 1) is based on the commercial airborne model Techpod, manufactured by HobbyUAV. It has a wingspan of 2.60 m and a fuselage length of 1.14 m. Its cruise speed is between 12 and 20 m/s and the maximum speed reaches 28.3 m/s. This UAV weighs 3.5 kg, including the payload and battery (1.25 kg without). The wings and fuselage were made from Expanded Polyolefin (EPO). The drone was powered with electrical propulsion: MT2216-V2 motor and two 5200 mAh 11.1 V LiPo batteries, controlled by an RCTimer 50 A electronic speed controller (ESC), that enabled two hours of endurance. No catapult was needed for take-off, and the plane was launched by hand. Characteristics of OVLI-TA are summarized in Table 1.

### 2.2. Wind Vector

Calculating wind vector is a complex task because it requires a large number of parameters, each of them needing to be measured very accurately. The velocity of the air with respect to earth V_w_ (wind) is the sum of the velocity of the aircraft with respect to earth V_g_ (ground speed), and the velocity of the air with respect to the aircraft V_air_ (airspeed):

(1) $$\vec{V_{w}}=\vec{V_{g}}+\vec{V_{\mathrm{air}}}$$

**Figure 2:**
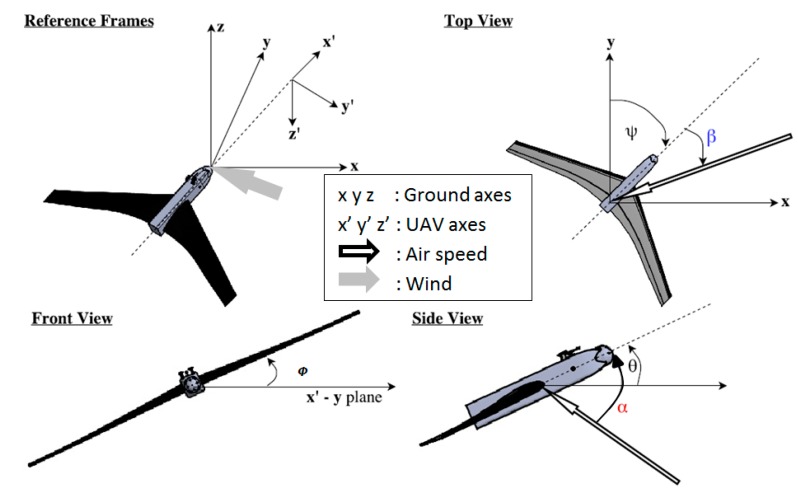
The earth and airplane coordinate systems were used for calculating the air velocity with respect to the earth referential. (x, y, z): earth coordinate system (i.e., east, north, upward); (x’, y’, z’): UAV coordinate system. The curved arrows indicate positive angles (see the text for angle definitions). Adapted from Reference [3].

The three components of ground speed (U_p_, V_p_, and W_p_ toward the east, north, and upward vertical directions, respectively) were provided by the GPS-IMU coupled system. The airspeed vector was first computed in the aircraft-related frame (x’, y’, and z’, see Figure 2), and then projected into the earth-related frame with the Euler angles: true heading *ψ*, roll angle *ϕ*, and pitch angle *θ*. In the aircraft-related frame, the three components of the airspeed V_air_ were calculated from the following three parameters: the velocity of the airflow V_a_, the attack (*α*), and sideslip (*β*) angles, with the following Equations [3]:

(2) $$V_{\mathrm{air}}=\{\begin{array}{c} -V_{a}D^{-1}\\ -V_{a}D^{-1}tan\beta \\ -V_{a}D^{-1}tan\alpha \end{array}$$

where D is expressed as:

(3) $$D=\sqrt{1+\tan\alpha^{2}+\tan\beta^{2}}$$

V_a_ is computed from the dynamic pressure ($P_{\mathrm{dyn}})$ measured with the Pitot tube, according to Bernoulli’s equation:

(4) $$V_{a}=\sqrt{\frac{2P_{\mathrm{dyn}}}{\rho }}$$

where ρ is the air density. This equation is based on incompressibility assumption, which is still valid given the airspeed of the UAS (~13 m/s). In the above equation, ρ is computed with perfect gas law, with the static pressure measured by the Pitot tube installed on the left side of the fuselage, and temperature measured in the hood as described below. The influence of humidity is not taken into account here.

The wind vector with respect to the earth reference was thus given as [3]:

U = −V_a_ D^−1^[sin*ψ*cos*θ* + tanβ(cos*ψ*cos*ϕ* + sin*ψ*sin*θ*sin*ϕ*) + tan*α*(sin*ψ*sin*θ*cos*ϕ* − cos*ψ*sin*ϕ*)] + U_p_ (5)

V = −V_a_ D^−1^[cos*ψ*cos*θ* − tanβ(sin*ψ*cos*ϕ* − cos*ψ*sin*θ*sin*ϕ*) + tan*α*(cos*ψ*sin*θ*cos*ϕ* + sin*ψ*sin*ϕ*)] + V_p_ (6)

W = −V_a_ D^−1^[sin*θ* − tanβcos*θ*sin*ϕ* − tan*α*cos*θ*cos*ϕ*] + W_p_ (7)

where U, V, and W (Up, Vp, and Wp, respectively) are the wind components (UAV groundspeed components) pointing toward east, north, and up, respectively. In the above equations, it is assumed that the airflow and inertial measurements were made at the same place, which is a reasonable assumption since the IMU is located inside the nose of the aircraft. For attack, sideslip, pitch, and roll angles, small angle approximations can often be made (in particular during straight and level portions of flight patterns), and hence the above equations can be simplified as [3]:

U = −V_a_ sin (*ψ* + β) + U_p_ (8)

V = −V_a_ cos (*ψ* + β) + V_p_ (9)

W = −V_a_ sin (*θ* − *α*) + W_p_ (10)

### 2.3. Autopilot and Acquisition System

**Figure 3:**
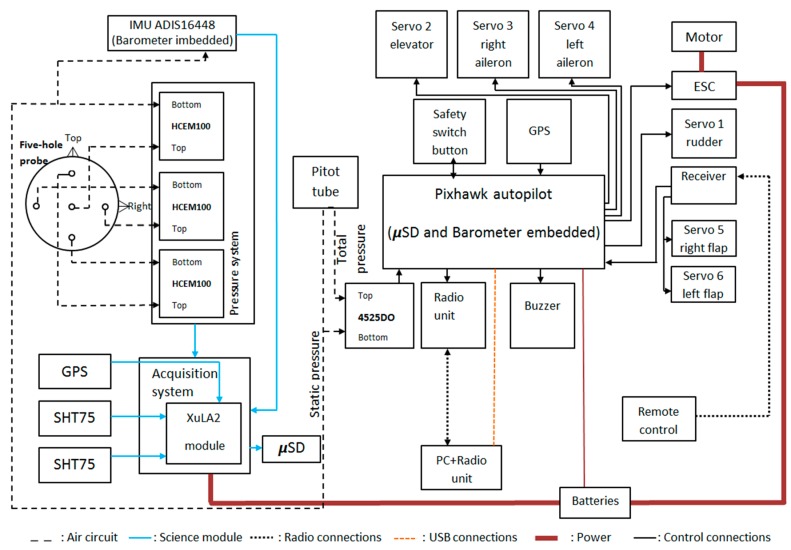
Diagram of connections between different devices on OVLI-TA.

OVLI-TA flies by the 3DR Pixhawk autopilot that ensures stable navigation. The autopilot was composed of an independent data acquisition system with a GPS for measuring the absolute position. This was coupled with the ADIS16448 IMU including a triaxial digital gyroscope, a triaxial digital accelerometer, and a triaxial digital magnetometer, in order to measure the three components of the angular speed, the linear acceleration, and the magnetic field, respectively. In addition, it contained a digital barometer from which the fast altitude variations can be computed, and an embedded temperature sensor. All these parameters were delivered to the acquisition system at a rate of 100 Hz. The GPS data was updated with a frequency of 5 Hz. The Pixhawk telemetry system sent in-flight data to the ground station, and also recorded them on an SD card. The data acquisition system is described in Figure 3. 

### 2.4. Meteorological Instrumentation of OVLI-TA

**Figure 4:**
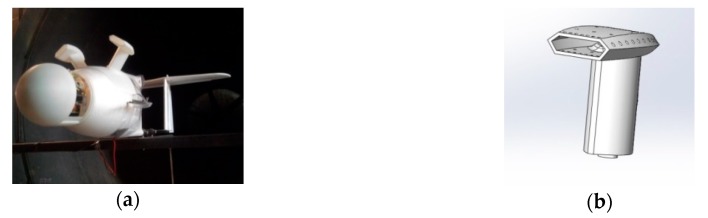
The hoods where the temperature and humidity sensors are placed: (**a**) hoods installed on the top of OVLI-TA fuselage; and (**b**) 3D design of the hoods.

OVLI-TA is equipped with two temperature and humidity sensors SHT75 (Sensirion), recorded at 2.5 Hz. They are placed inside dedicated hoods (Figure 4). These 3D printed hoods were designed in such a way that droplets (if any) go straightforward by inertia through the inlet without any contact with the sensor, whereas the measured air parcels were deflected inside the body of the hood with a stable velocity relative to the sensor.

**Figure 5:**
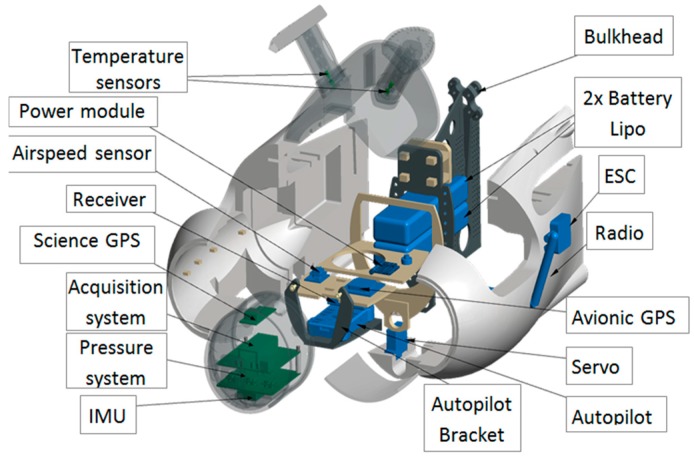
Exploded diagram of the electronic devices on OVLI-TA.

The attack and sideslip angles were computed from differential pressures according to the system proposed in 1983 by Brown et al. [18] on a piloted airplane. A 3D printed five-hole hemispherical probe was situated on the nose of the drone (see Figure 4), the holes were connected to three differential pressure transducers (HCEM010, sampled at 100 Hz) by silicone tubes. There were five pressure ports: the center hole was in the longitudinal axis of the airplane, the four others were placed above and below, and to the right and left of the central hole, making an angle of 45° to the center of the sphere. The diameter of the hemisphere and holes were 10 cm and 1 mm, respectively. The first pressure sensor made it possible to measure the difference between the total pressure (from the center hole of the five-hole probe) and the static pressure (from the Pitot tube) in order to calculate the dynamic pressure and thus the airspeed, as a redundant system to the Pitot tube. The other sensors measured the differential pressure between the up and down (resp. left and right) holes in order to calculate the angle of attack α (resp. angle of sideslip β). These sensors need a calibration procedure (see below). The acquisition rate of pressure data was 100 Hz. The payload was installed at the front of OVLI-TA. The overall equipment is presented in Figure 5.

### 2.5. The Five-Hole Probe Calibrations

The wind tunnel test for the five-hole probe calibration was conducted at the IMFT (Institut de Mécanique des Fluides de Toulouse, Toulouse, France) in 2014. The range of wind speed produced by the wind tunnel was from 1 to 30 ms^−1^, and the section of the tunnel was 2.4 m × 2.0 m.

**Figure 6:**
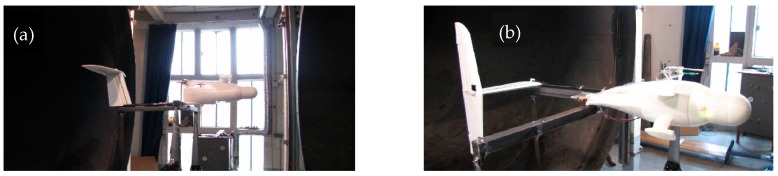
OVLI-TA during the wind tunnel test: (**a**) angle of attack testing; and (**b**) angle of sideslip testing [19].

The data acquisition system was in the same configuration as during flights. Figure 6 shows how the UAV was installed in the wind tunnel. 

**Figure 7:**
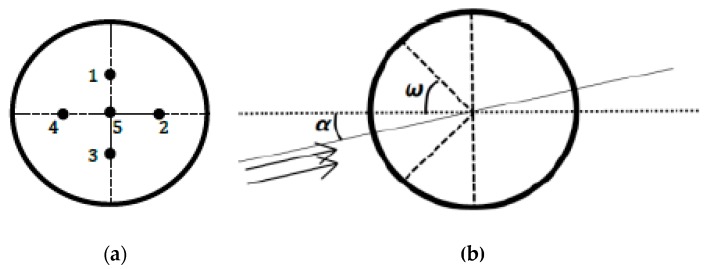
The five-hole probe; (**a**) front view; and (**b**) right view.

Theoretically, and according to Brown et al. [18], the equation for the attack angle α is:

(11) $$\hat{\Delta P_{\alpha }}=\hat{P_{1}}-\hat{P_{3}}=-\frac{9}{4}*\sin2\omega *\sin2\alpha ,$$

where $\hat{P_{1}}$ and $\hat{P_{3}}$ are the pressures at port 1 and 3 of the five-hole probe corresponding to the holes above and below the central hole, respectively, normalized by the dynamic pressure; and ω is the angle between the longitudinal axis of the 5-hole probe and the axis passing through one port and center of the sphere (Figure 7). The same equation stands for sideslip angle, replacing α with β and ports 1 and 3 with ports 4 and 2, respectively. Therefore, by assuming low values for the attack and sideslip angles, and given that ω=π/4, Equation (11) can be rewritten as:

(12) $$\alpha =k_{\alpha }^{-1}*\hat{\Delta P_{\alpha }}\;\beta ={k_{\beta }}^{-1}*\hat{\Delta P_{\beta }},$$

with a theoretical value of $k_{\alpha }$ = $k_{\beta }$ = 4.5 rad^−1^ (0.0785 deg^−1^)

In fact, $k_{\alpha }\mathrm{and}k_{\beta }$ can depart from the theoretical value for several reasons; like a non-perfect hemispherical shape of the nose, a shift in the position of the holes with respect to the theoretical position, or a pressure perturbation related to the fine shape of the pressure ports. The exact value of the coefficients was therefore determined from wind tunnel tests. The airplane was fixed on a structure, the pitch of which was varied and measured. The principle of the calibration was to consider that in the wind tunnel flow, when the roll is zero, the pitch and attack angle must be identical. Similarly, when the airplane was rolled by 90°, the pitch of the structure was equal to the sideslip angle.

**Figure 8:**
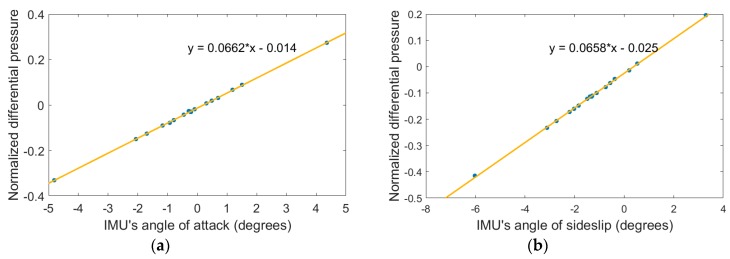
(**a**) Left plot: normalized differential pressure as a function of attack angle; and (**b**) right plot: normalized differential pressure as a function of sideslip angle.

The angle of attack varied between −5° and 5°, with a zero angle of sideslip, so as to determine the sensitivity factor $k_{\alpha }$. The relationship between the differential normalized pressure and IMU’s angle of attack is shown in Figure 8a. From this plot we deduced that the sensitivity factor of the attack angle was $k_{\alpha }=0.0662$ deg^–1^. Similarly, the angle of sideslip varied between −5° and 5° by maintaining the angle of attack equal to zero, in order to determine the sensitivity factor $k_{\beta }$. The relationship between the differentially normalized pressure and the angle of sideslip was $k_{\beta }=0.0658$ deg^–1^ as shown in Figure 8b.

### 2.6. Flight Sites and Patterns

**Figure 9:**
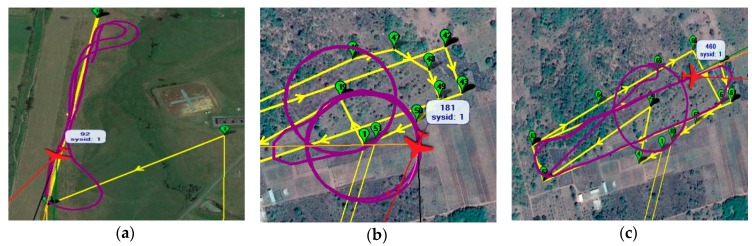
Flight patterns: (**a**) Flight conducted in Lannemezan site. The 60 m tower is situated around 200 m to the south of the lower left corner of the picture (length of straight flight: around 500 m); (**b**) flight conducted in DACCIWA site on June 29, 2016 (Radius circle: 100 m); and (**c**) flight conducted in DACCIWA site on July 15, 2016 (radius circle: 100 m; and length of straight flight: 700 m).

OVLI-TA flew on two different sites. Firstly, flight tests were conducted on March 24, 2016 in Lannemezan, in the south of France. This site was the instrumental platform of the “Laboratoire d’Aérologie”, belonging to the Pyrenean Platform for the Observation of the Atmosphere (P2OA, p2oa.aero.obs-mip.fr). A 60 m tower was equipped at three levels (30 m, 45 m, and 60 m) for turbulence measurements with sonic anemometers that gave us the three wind components and the so-called sonic temperature at a frequency of 10 Hz. In addition to that, relative humidity and temperature were measured at these levels, and recorded at 1 Hz. The flight pattern chosen was a succession of straight and level paths back and forth, which was the most appropriate type of flight plan for atmospheric turbulence measurements (Figure 9a). Each straight line of flight was 300–400 m long and lasted around 30 s; hence 61 sequences of flight in a straight line were selected and used for turbulence calculation. OVLI-TA flew for almost 1 h, between 60 m and 130 m but not exactly in the footprint of the tower. 

Afterwards, the drone participated in the DACCIWA campaign in West Africa (Savè, Benin). The site was equipped with radio soundings, remotely piloted airplane system, cloud camera, cloud radars, Doppler lidar, radiometer, sodar, UHF wind profiler, and meteorological/turbulence/chemistry ground stations. The ground-based field campaign took place from June 14 to July 30, 2016 [20]. We chose to present results from the flights on June 29, 2016 and July 15, 2016. On June 29, 2016, the drone flew between 10 m and 700 m agl, with ascending and helical patterns (Figure 9b) in order to compare OVLI-TA profiles of wind, temperature, and humidity with those obtained with radiosondes regarded as a reference. On July 15, 2016, the OVLI-TA flight pattern consisted of straight and level runs of similar lengths as shown in Figure 9c. The drone flew between 10 m and 1000 m agl, with ascending helices and descending straight lines.

## 3. Results

### 3.1. Mean Wind and ABL Profiles

**Figure 10:**
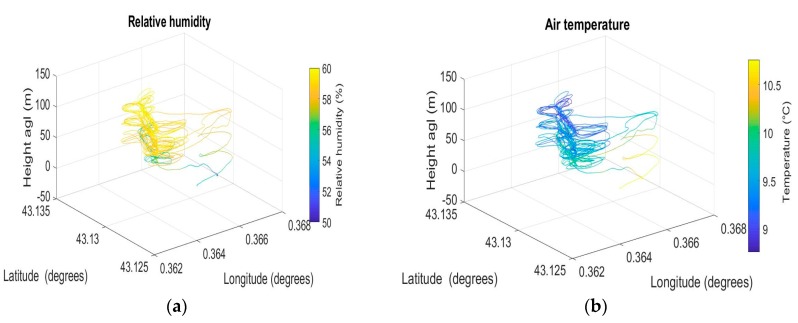

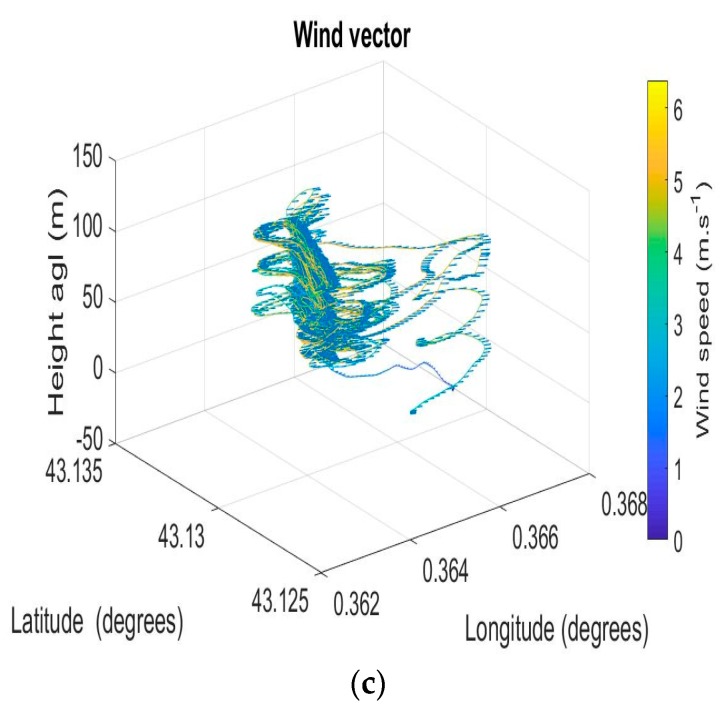
Relative humidity (**a**), temperature (**b**), and wind speed (**c**) measured by OVLI-TA during a flight in Lannemezan on 24 March 2016. The time window is from 12:35 to 13:27 (UTC).

The 3D representation (Figure 10) of humidity, temperature, and wind vector provides an overview of the full flight pattern in Lannemezan. Temperature and humidity appeared consistent during the flight, with little variation along each individual straight leg. However, the altitude range analyzed was not wide enough to extract the variations, which should be observed on vertical profiles. That was the reason why we focused the analysis of this flight on wind and turbulence parameters, the temperature and humidity profiles being analyzed from the DACCIWA flights (see later).

As mentioned above, wind (speed and direction) was among the most critical parameters extracted from airplane measurements, because it required a lot of variables to be measured (see previous Equations (5)–(7)). Furthermore, the parameters to be computed (wind components) were generally one order of magnitude lower than some variables in the equations, like the groundspeed or airspeed components. The resulting errors could thus be considerable, and evolve with the orientation of the airplane with respect to the wind’s direction. For example, overestimating true airspeed leads to an underestimation (resp. overestimate) of wind speed when the airplane is facing the wind (resp. has the wind at its back), whereas the impact on the wind direction estimates is much lower. On the other hand, when the airplane is flying crosswind, an error on the true airspeed impacts (at the first order) the estimate of the wind direction, whereas the wind speed estimate is biased through an error on the sideslip angle. If one assumes that the mean wind does not vary much during a back and forth sequence, one can thus extract information about the bias of those variables from the difference in the wind computed on the two paths. These biases can be computed from appropriate flight sequences (like back and forth runs), and the quality of the wind estimates thus improved. 

**Figure 11:**
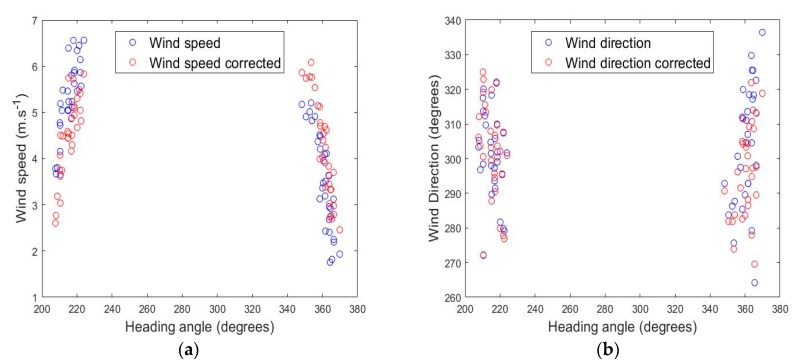
Wind speed (**a**) and wind direction (**b**) as a function of heading angle, estimated on the 61 flight sequences before (in blue) and after (in red) the bias correction.

In the flight presented on Figure 9, OVLI-TA flew 61 straight sequences over the same 500 m long ground track, with true headings around 210° and 360° (hereafter called “SSW runs” and “N runs”, respectively), at heights between 60 and 130 m. The mean wind speed and direction was computed on each of these sequences, and the global scatter of the results was analyzed. (Thereafter, the terms “mean wind direction”, that will be used several times for simplicity, represents the direction calculated from the mean wind components in an earth-related frame). Figure 11 presents the mean wind speed and direction for the 61 sequences according to the heading of the drone. Without any adjustment, it can be remarked that the cluster of SSW runs showed wind speed values higher on average than the cluster of N runs. The difference was less significant on the wind direction. Taking into consideration this flight configuration and once a correction (an offset equal to 0.055 rad) had been applied to the sideslip angle, the two clusters of wind direction get comparable shapes. The impact on the wind direction was weaker, with a reduction in the overall range of the computed values (from [263°–337°] without correction to [269°–327°]). The origin of this offset probably lies in a misalignment between the IMU and the five-hole probe, due to the successive mounting and dismounting of the system on the airplane after the wind tunnel calibration. 

The improvement brought by the correction was evaluated by comparing the standard deviation of the wind speed and direction before and after correction (Table 2). Wind direction is a circular variable that requires a specific method for estimating its standard deviation, because we have to cope with the passage at 0/360° (or +/− 180°). We used the method described by Yamartino [21], who proposed the following relations:

***σ_P_*** = sin^−1^(***ε***)*[1 + 0.1547*ε^3^] (13)

***ε*** = 1 − (S_a_^2^ + C_a_^2^) (14)

S_a_ = n^−^^1^Σ_i_sin ***P***_i_ (15)

C_a_ = n^−1^Σ_i_cos ***P***_i_ (16)

where ***σ*** is the standard deviation of wind direction, and ***P***_i_ is the wind direction on the ith of the 61 legs.

The values in Table 2 confirm that the correction applied to the sideslip angle had a major impact on the wind speed estimate, with a standard deviation reduced by 25% (from 1.31 m/s to 0.98 m/s) and a minor impact on the wind direction estimate by reducing the standard deviation by only 8% (from 15.0° to 13.8°). The flight configuration was close to the “cross-wind” one, and the adjustment on the sideslip angle had a direct impact on the wind speed estimate. The overall performance was regarded as very satisfactory for the wind estimate with a mobile platform. 

**Figure 12:**
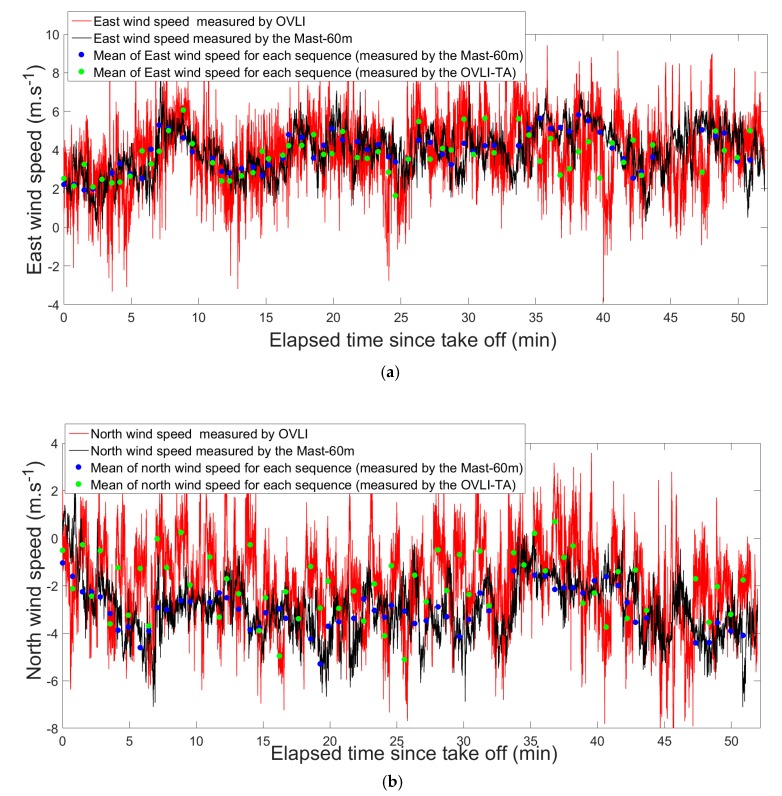
Continuous lines: north and east wind speeds measured by OVLI-TA (in red) and on the tower at 60 m (in black), during the whole flight. Dots: mean of north and east wind speeds for each straight and level sequence of the flight measured by OVLI-TA (in green), and on the tower at 60 m for the same periods of time (in blue).

Once optimized, thanks to the flight pattern, the north and east wind speeds measured by OVLI-TA were compared to those measured by the sonic anemometer mounted at the 60 m level on the tower (Figure 12). For each platform, we present two time series: the instantaneous values, which contained the turbulent fluctuations; and values averaged over the straight and level runs of OVLI-TA, and averaged over the same corresponding periods on the tower. On the instantaneous time series, we observed that the two measurements were consistent, even though the OVLI-TA signal presents a higher variability, related in part to poorer wind estimates during half turns between two straight flights; in part due to the altitude range flown by the drone (between 60 and 130 m, whereas the tower observations are made at 60 m only), and in part to the difference between the areas sensed by the two platforms (given the wind direction, the footprint of the tower lies to its WNW, whereas the flight track is to the north of the tower). Furthermore, if we look at the values averaged over the periods of time of the straight sequences (green and blue dots on Figure 12), we find a very satisfactory agreement, given the scatter induced by the difference between the two platforms regarding their footprint, and the altitude of air masses sensed. 

The absolute accuracy of the sensor used in the tower was lower than 0.1 m/s (https://www.campbellsci.com/csat3), i.e., an order of magnitude lower than what can be expected with a mobile platform like a drone. For each of the 61 samples (blue and green dots in Figure 12), we therefore compared the east and north mean components between the drone and tower. For each component, the difference between the two platforms was quantified through the mean absolute error (MAE) and the standard deviation of the difference computed on the 61 values. MAE is defined as: $\mathrm{MAE}=\overset{n}{\underset{i=1}{\sum }}\frac{\left|U_{Mast,i}-U_{OVLI,i}\right|}{n}$.

Table 3 summarizes the obtained results. The order of magnitude of these errors was 1 m/s. Though the standard deviation of the difference between the two platforms was identical for the two components, we observed a higher MAE on the north component (1.2 m/s) compared to that on the east component (0.8 m/s). We propose two hypotheses to explain such a difference: first this could be due to the distinct local areas of where the 60 m tower is situated and where the drone was flying, because OVLI-TA was not exactly flying in the footprint of the 60 m tower (the drone tracks are some hundreds of meters away from the tower) and the levels of flight were between 60 m and 130 m (whereas the tower observations are at 60 m only); second, a bias might remain in the drone north wind estimate resulting from a non-perfect calibration of a parameter (in the present case, given that the aircraft headings are close to the N or S orientations, the true airspeed might be suspected). However, we considered that these errors belong to the acceptable range of the errors targeted for the wind estimates with our mobile platform, and must be regarded as the upper bound of the drone error.

**Figure 13:**
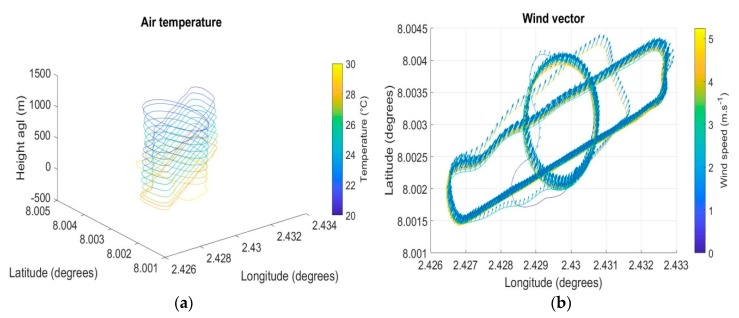
Air temperature (**a**) and wind vector (**b**) measured on July 15, 2016 at 16:45 (UTC).

After flights in Lannemezan, OVLI-TA was operated during the DACCIWA campaign for profiling temperature, humidity, wind speed, and direction in the ABL between 10 and 1000 m agl. Figure 13 illustrates the observations of temperature and wind during the flight on July 15, 2016 that started at 16:45 UTC and lasted for 40 min. Stacked back and forth sequences were made on a racetrack pattern, and a continuous profile was obtained from a helical pattern.

**Figure 14:**
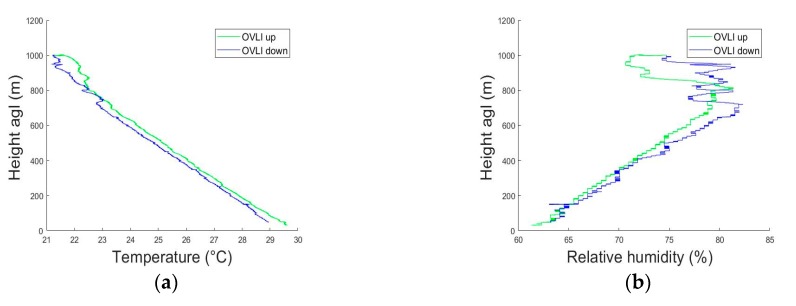

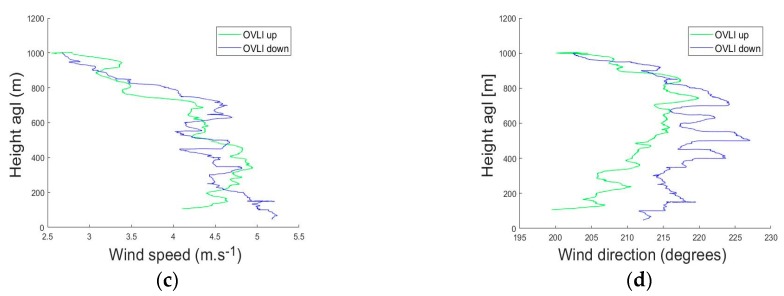
Profiles of temperature (**a**), relative humidity (**b**), wind speed (**c**), and wind direction (**d**) measured during ascending (in green) and descending (in blue) flight sequences on July 15, 2016.

The quality of the measurements was checked by comparing profiles during the ascending and descending parts of the flight. This is shown in Figure 14, for the wind speed and direction, relative humidity, and temperature. The differences in relative humidity below 600 m were within the expected accuracy of the sensor. The large fluctuations above 600 m were related to the so-called entrainment zone, which lies in the upper part of the ABL, and contains a mixture of air parcels coming from the well-mixed layer below, and from the upper layer above, the latter being characterized by much warmer and drier conditions. The difference in the two temperature profiles was also the highest in this area, for the same reasons. In the mixed layer below, the temperature difference between the two profiles was of the order of 0.2–0.3 °C in the 100–600 m layer, and could reach 0.5–0.6 °C in the 50–100 m layer, which was also within the absolute accuracy of the sensor. Note that on this flight, the ascent speed of OVLI-TA was 1.5 m/s and the descent speed was 0.7 m/s. 

**Figure 15:**
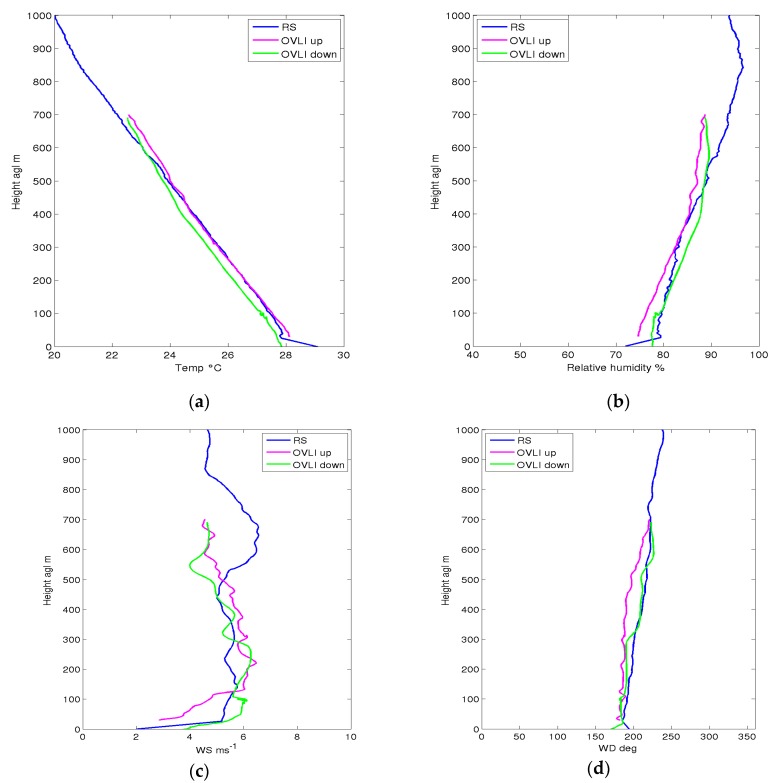
Same as Figure 14, but for the flight on 29 June 2016 and together with the observations obtained from a radiosonde (RS). (**a**) Temperature (°C), (**b**) relative humidity (%), (**c**) wind speed (m.s^−1^), (**d**) Wind direction (degrees). Lines: radiosonde measurements (in blue), measurements of OVLI-Ta during ascent (in pink) and descent (in green).

During the campaign, radiosondes were launched under meteorological balloons. This system measured the profiles of temperature, relative humidity, and wind. The ascending speed of the radiosonde was about 5 m/s, the 1st kilometer of the atmosphere was therefore sensed in around 200 s, which can be considered as “instantaneous” with regard to atmospheric variability. Radiosondes are generally considered as a reference measurement system, though moisture observations were questioned for specific conditions. During the OVLI-TA flight of June 29, a radiosonde was launched at 16:59 UTC, whereas two profiles (ascending and descending) were made by the drone between 17:12 and 17:35 UTC. The comparison of the profiles measured by the two systems is shown in Figure 15. The radiosonde used (http://www.meteomodem.com/docs/fr/Brochure-m10.pdf) had a time resolution of 1 s, and the response time of the sensors was 1 s for temperature and 2 s for humidity; consequently we can expect a vertical delay in the order of 5 m for temperature and 10 m for humidity. Due to this delay, and given the vertical gradients observed in the ABL, the profiles were therefore biased by ~+0.04 °C for temperature and ~−0.3 % for relative humidity. Such values were low enough to consider the radiosonde profiles as the reference against which the drone profiles have to be compared. The agreement was good for the three parameters; the main discrepancy being observed was in the 500–700 m layer, humidity, and wind speed/direction. However, we observed a difference between ascending and descending profiles which could be due to sensor response time. This can be estimated from the value of difference and the ascending and descending speeds during the profiles. To do that, we focused on altitude ranges chosen in the so-called “mixed layer”, i.e., above the atmospheric surface layer where the vertical and horizontal homogeneity is large, and well below the top the ABL where strong vertical gradients exist. For the retained flight sequences, only 12 min elapsed between the start of the ascent and the end of the descent, so we can assume a steady-state situation. The response time $\tau_{x}$ is thus computed as:

(17) $$\tau_{x}=\frac{x_{up}-x_{down}}{\left(\frac{dx}{dz}\right)\left(v_{up}-v_{down}\right)},$$

where *x* being either the temperature T or relative humidity RH. The indexes up and down refer to the ascending and descending phases, respectively; *v* is the vertical speed of the drone; and *z* is the altitude. *v_up_* and *v_down_* were 1.4 and −5.0 m/s, respectively. The resulting response times were 8.9 s and 14.8 s for T and RH, respectively. The corresponding delays in the observed profiles during the ascent phases were therefore ~12 m and ~21 m for temperature and relative humidity, respectively. Such values were around twice those computed for the radiosonde, considered as the reference, and the corresponding biases were +0.10 °C for temperature and −0.6 % for relative humidity, which lay well within the absolute accuracy of the sensors. This means that one can easily cope with the biases resulting from the response time of the sensors provided that the ascending or descending speed of the drone is low enough.

### 3.2. Turbulence

**Figure 16:**
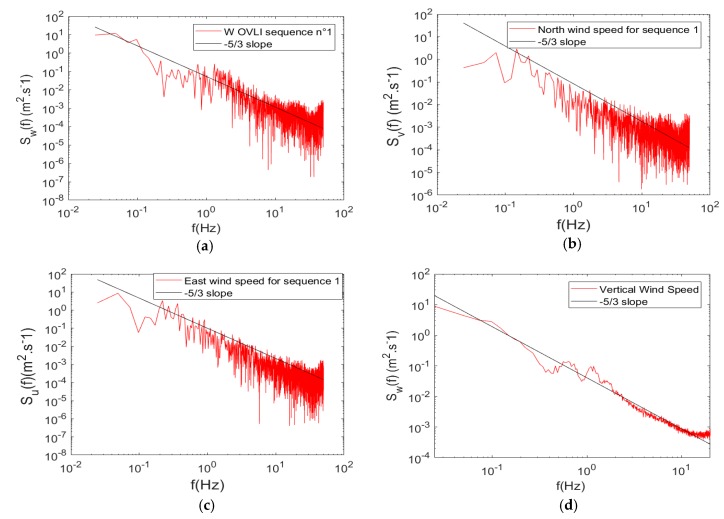
(**a**) Spectra of vertical wind speed, (**b**) north wind speed, (**c**) east wind speed, and (**d**) mean spectrum of vertical wind speed for all sequences. Note that the frequency scale is different on (**d**).

The flight on the Lannemezan site was suitable to investigate the capability for turbulence observation. The three wind components were computed at a rate of 100 Hz. In this section, we investigate the maximum frequency at which the signals can be used. Figure 16a–c shows the energy spectra of the north (V), east (U), and vertical (W) wind components, for a straight and level sequence. Given the meteorological conditions, and that the flight was performed in a convective boundary layer, a 3D turbulence was expected, with a −5/3 power slope in the inertial sub-range. This behavior was well observed on the spectra, except for the highest frequencies where the signal was noisy. We cannot investigate in detail the possible (and multiple) origins of this noise. Such a shape on spectra is frequently observed on wind signal computed with aircraft platforms, because a flying system constitutes an adverse environment for fine and high-frequency measurements. In order to improve the statistics, we averaged the individual energy spectra computed on 61 straight and level sequences. An identical period of time was retained on each sequence, in order to have the same frequencies on the individual spectra. The resulting spectrum for the vertical wind was thus presented (up to 10 Hz) in Figure 16d. We highlight the result on the vertical wind, because this component was involved in the estimate of the vertical turbulent fluxes through the eddy–covariance technique, as the horizontal turbulent fluxes were much lower in the ABL. The −5/3 slope, characteristic of the inertial sub-range, was observed on this spectrum up to 10 Hz. 

**Figure 17:**
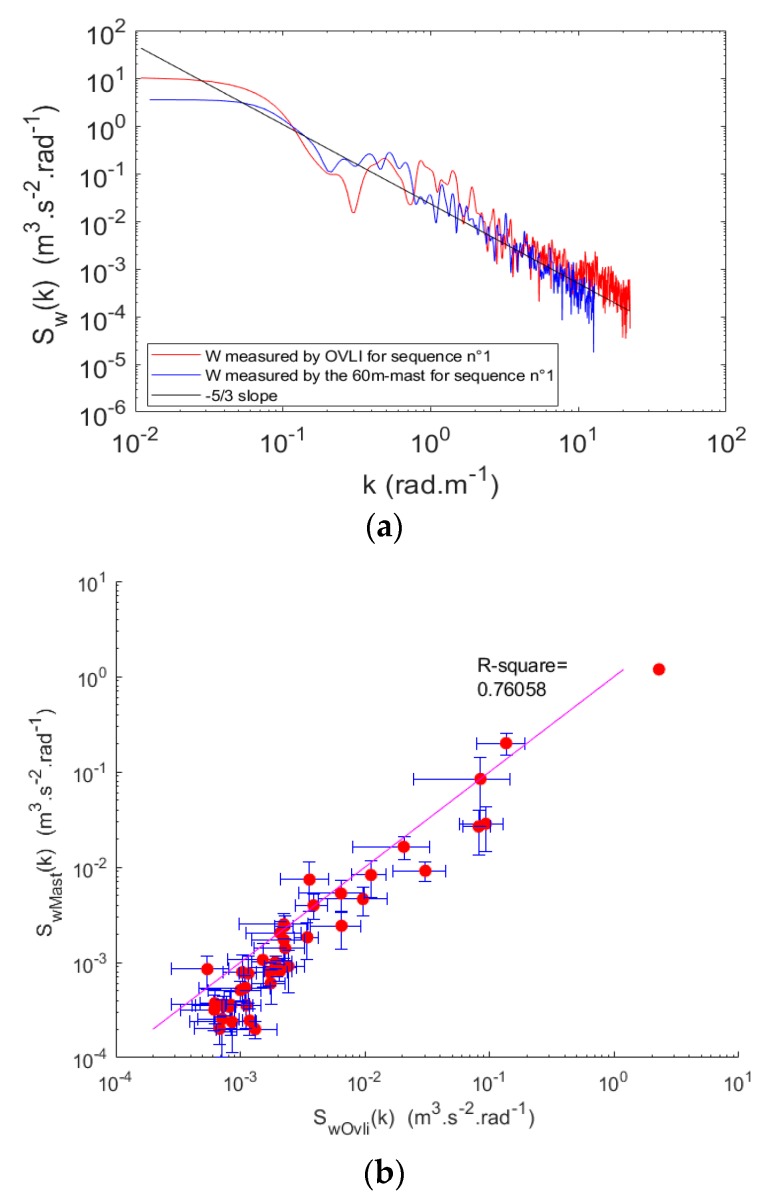
(**a**) Vertical wind spectrum as a function of wavenumber computed during the first straight sequence. OVLI-TA measurements are in red and the corresponding 60 m tower in blue. The spectra are computed using Welch’s method. (**b**) The scatter plot of the power density of the mast against OVLI-TA.

In order to go further into the qualification of turbulence measurements, we made a comparison with the 60 m tower observations. As mentioned above, there was a sonic anemometer installed at the top of the tower, measuring the three wind components at a rate of 10 Hz. We focused on the vertical wind, for the reasons mentioned above. In order to compare between tower and drone spectra, we must go back to spatial scales as observed by the two platforms. In other words, we must convert a frequency f into the corresponding wavenumber k assuming Taylor’s hypothesis k = 2*πf/u*, where u is the mean speed of the flow with respect to the sensor. Hence, we considered the mean airspeed for OVLI-TA, and the mean wind speed for the tower. Figure 17a shows the comparison for the first sequence of straight flight using the Welch periodogram. The period of time T_t_, over which the tower observations were considered is defined according to the distance in the air mass sampled by the drone during this sequence: T_t_ = T_d_ × V_a_ × V_w_^−1^, where V_a_ is the mean true airspeed measured by the drone, T_d_ is the duration of the drone path, and V_w_ is the mean wind speed measured on the tower. Given the differences between the two air masses sampled by the two platforms, the spectra cannot be identical. However, the general shape and the characteristic scales should be similar. This is confirmed in Figure 17a,b where it can be seen that the two platforms capture the same level of energy, even in the inertial sub-range, up to wavenumbers of 4–5 rad/m. This means that the drone was able to correctly sample the ABL down to spatial scales of the order of 1 m, i.e., was able to capture the scales, which significantly contribute to the turbulent kinetic energy.

A scatter-plot provides a more in-depth comparison of the two spectra. However, since the Taylor’s hypothesis is used to convert the frequency spectra into wavenumber spectra, and given that flow speeds used for this conversion are different for the two platforms (the true airspeed for the aircraft and the wind speed for the tower), the wavenumbers at which the spectral energy is computed are different. Therefore, the energies cannot be directly compared. To cope with this difficulty, we computed for each platform the average energy (on the Welch spectra) in non-overlapping wavenumber ranges identical for the two platforms. Furthermore, the scatter was quantified with the standard deviation in each wavenumber bin. The result is presented in Figure 17b. The agreement was quantified through the determination coefficient (0.76). It can be seen that the energy computed agreed well between the two platforms, except at the lowest energies for the aircraft where the values were higher. This is related to the noisy tail of the aircraft spectrum, as mentioned previously.

## 4. Discussion

The development of the small, unmanned aerial system OVLI-TA for probing the ASL/ABL makes it possible to explore atmospheric layers as a complementary method to towers and other platforms. OVLI-TA is easy to operate, in a category of UASs for which the air traffic rules remain acceptable, avoiding the constraints we have to deal with for heavier airplanes. Nevertheless, the payload capacity (~2 kg) allows us to embark high-performance instrumentation, composed of atmospheric sensors, an inertial platform, and an efficient autopilot. This instrument package has revealed its capacity to measure the temperature, humidity, and wind components all along the aircraft’s trajectory. In particular, the three wind components, although hard to compute on mobile platforms, can be estimated not only for their mean value, but also for the turbulent fluctuations. In particular, the instantaneous values of the airspeed vector, required for these calculations, are computed from a multi-hole probe installed on the nose of the airplane, and calibrated in a wind-tunnel for the estimates of attack and sideslip angles.

The quality of the measurements was determined from self-consistency methods and also by comparison with other measurements on platforms considered as a reference. For temperature and humidity profiles, we checked the similarity between the observations collected during ascending and descending flight profiles, and compared the observations to those obtained with radiosonde profiles. From the difference between up- and down-ward profiles performed in a short time range, we estimated the response times of the temperature and relative humidity probes, as around nine and 15 s, respectively. With a profiling vertical speed around 1 m/s, this allows us to lower the bias of the observed profiles at a level twice that of the radiosonde under meteorological balloons. For wind speed and direction, the variability of the estimates during back and forth runs, allowed us to adjust calibration coefficients and reduce the scatter of the estimates. Comparison with the 60 m tower observations revealed an excellent agreement, even though the air mass sensed by the two platforms was not identical. The absolute accuracy of the wind components is better than 1 m/s, because this value, deduced from the difference between drone and tower estimates, involves at least in part the atmospheric variability. The turbulence spectra, computed on straight and level flight sequences, revealed the expected slope in the inertial sub-range, up to a frequency of about 10 Hz. We compared these spectra with those computed from the tower observations, once the transformation of frequency into wavenumber had been done according to the sample velocity of each platform. The spectra agree as well as it can be expected, given the difference between the footprints of the two platforms. In addition, a scatter plot of power density of the mast against OVLI-TA confirms that the agreement is good up to 10 Hz.

The OVLI-TA platform is able to sample ABL turbulence with a spatial resolution of around 1 m. We can question whether this allows us to capture most of the energetic turbulence. In fact, 3D turbulence spectra in the ASL, when represented in the unit of variance, are characterized by a peak occurring at a certain frequency, with the energy decreasing on each side of the peak [22]. We can consider that the turbulence observation is appropriate when the sampling frequency of the measures is at least one order of magnitude higher than that of the peak. Starting from the surface, this peak shifts continuously towards lower frequencies (larger wavelengths) when the observation height increases, because the size of eddies grows with the distance from the surface. For convective, daytime conditions, we can expect a peak wavelength of the order of 3–5 z, where z is the height above the ground [22,23]. That means that at z = 10 m, which is the lowest height at which the drone could be reasonably operated, we are able to resolve eddies of a size 30–50 times smaller than the most energetic ones, and therefore most of the significant turbulence energy can be captured.

## 5. Conclusions

OVLI-TA is a small affordable UAV, developed in France. It was instrumented for profiling the ABL and measuring turbulence. It flew in two different sites, Lannemezan (France) and Savé (Bénin). From the comparison between OVLI-TA and respectively the 60 m tower and “radiosondes” measurements, we demonstrated its efficiency in measuring the turbulence up to 10 Hz, which is equivalent to a horizontal resolution of around 1 m. In spite of the quite slow temperature and moisture sensors, the UAV is able to give reliable profiles in the ABL provided that the vertical speed of the aircraft is kept slow (~1 m/s).

In the near future, we plan to extend OVLI-TA’s capabilities with fast response temperature and moisture sensors. This will give us the possibility to compute kinematic heat and moisture fluxes, which are essential terms in ABL dynamics. Furthermore, we are developing a new instrumentation on a heavier drone, to extend the payload to 5 kg and the endurance to 5–6 h. This will allow us to embark high-performance instruments, in particular for turbulence measurements, and to fly in more adverse conditions, such as strong winds and those with high turbulence. However, the operation of this new system would be more restrictive, especially with regard to flight authorization and operation. We will therefore benefit from the complementarity of the two systems. 

## Figures and Tables

**Table 1:** Technical characteristics of OVLI-TA.

Wingspan/Wing Area	Fuselage Length	Dry Weigh/Payload	Max Cruise Speed	Ascent Range	Endurance
2.6 m/0.4 m^2^	1.14 m	1.25/2.25 kg	28.3 m/s	3–10 m/s	1–2 h

**Table 2:** Standard deviation of corrected and non-corrected values of wind speed and wind direction computed on the 61 flight sequences by OVLI-TA.

Wind Speed (m/s)		Wind Direction (Degrees)	
Non-Corrected	Corrected	Non-Corrected	Corrected
1.31	0.98	15.0	13.8

**Table 3:** Mean absolute error of east and north wind speed. Standard deviation of the difference between east (or north) wind component measured respectively by the 60 m mast and OVLI-TA.

	East Wind Component (m/s)	North Wind Component (m/s)
MAE	0.8	1.2
***σ*** (U_Mast_-U_OVLI_)	1.0	1.0
